# The efficacy and safety of different endovascular modalities for infrapopliteal arteries lesions: A network meta-analysis of randomized controlled trials

**DOI:** 10.3389/fcvm.2022.993290

**Published:** 2022-11-09

**Authors:** Julong Guo, Yachan Ning, Hui Wang, Yu Li, Zhixiang Su, Fan Zhang, Sensen Wu, Lianrui Guo, Yongquan Gu

**Affiliations:** ^1^Department of Vascular Surgery, Xuanwu Hospital, Capital Medical University, Beijing, China; ^2^Department of Intensive Care Medicine, Xuanwu Hospital, Capital Medical University, Beijing, China; ^3^Department of General Surgery, Xuanwu Hospital, Capital Medical University, Beijing, China

**Keywords:** endovascular procedures, critical limb ischemia, chronic limb-threatening ischemia, peripheral arterial disease, infrapopliteal artery, network meta-analysis

## Abstract

**Background:**

Endovascular treatment has become the first-line therapy for infrapopliteal artery occlusive disease (IPOD), while the optimal endovascular method remains to be determined. We performed a network meta-analysis (NWM) of randomized controlled trials (RCTs) to simultaneously compare the outcomes of different endovascular modalities for IPOD.

**Methods and results:**

The Pubmed, Embase, and Cochrane databases were used as data sources. The NWM approach used random-effects models based on the frequentist framework. In total, 22 eligible RCTs (44 study arms; 1,348 patients) involving nine endovascular modalities or combinations [balloon angioplasty (BA), drug-coated balloon (DCB), drug-eluting stent (DES), atherectomy device + BA (AD + BA), AD + DCB, balloon-expandable bare metal stent (BMS), self-expanding stent (SES), absorbable metal stents (AMS), and inorganics-coated stent (ICS)] were included. BA had a lower 12-month primary patency rate than DCB (RR 0.50, CI 0.27, 0.93) and AD + DCB (RR 0.34, CI 0.12, 0.93). AD + DCB decreased 6-month TLR compared with AMS (RR 0.15, CI 0.03, 0.90), and DES decreased it compared with BMS (RR 0.25, CI 0.09, 0.71). DCB had a lower 6-month TLR rate than AMS (RR 0.26, CI 0.08, 0.86) and BA (RR 0.51, CI 0.30, 0.89). BA had a higher 12-month TLR rate than DCB (RR 1.76, CI 1.07, 2.90). According to the value of the surface under the cumulative ranking curve (SUCRA), AD + DCB was considered the best treatment in terms of primary patency at 6 months (SUCRA = 87.5) and 12 months (SURCA = 91). AD + BA was considered the best treatment in terms of 6-month TLR (SUCRA = 83.1), 12-month TLR (SURCA = 75.8), and 12-month all-cause mortality (SUCRA = 92.5). In terms of 12-month major amputation, DES was considered the best treatment (SUCRA = 78.6), while AD + DCB was considered the worst treatment (SUCRA = 28.8). Moreover, AD + BA always ranks higher than AD + DCB in the comparison including these two combinations. Subgroup analyses of modalities without stenting did not significantly change the primary outcomes.

**Conclusion:**

ADs showed noteworthy advantages in multiple terms for IPOD except for 12-month major amputation. AD + BA may be a better method for IPOD than AD + DCB. The efficacy and safety of ADs are worthy of further investigation.

**Systematic review registration:**

[https://www.crd.york.ac.uk/prospero/], identifier [CRD42022331626].

## Introduction

Infrapopliteal artery occlusive disease (IPOD) is the most common cause of critical limb ischemia (CLI), which is associated with considerable morbidity and mortality (1). If left untreated, it can lead to increasing pressure on the quality of life and the economy. A more effective and safe treatment option is continuously being explored to relieve the ischemic pain, promote wound healing, salvage the limbs, and achieve amputation-free survival (2). With the emergence and development of endovascular technology, endovascular treatment of IPOD has achieved comparable efficacy and lower perioperative morbidity and mortality compared with open surgery and has become a first-line treatment for IPOD (3).

Deployment of plain balloon angioplasty (BA) with bailout bare metal stent has long been considered to be the standard endovascular treatment of IPOD (3, 4). As a traditional endovascular modality of treating IPOD, BA was found to be associated with high rates of restenosis and reintervention due to elastic recoil, neointimal hyperplasia, and vascular remodeling (5), which has actively driven the innovation and application of newer equipment, such as drug-coated balloons (DCBs), drug-eluting stents (DESs), and atherectomy devices (ADs) (6–8). Concomitantly, some randomized controlled trials (RCTs) have been performed to compare the efficacy and safety of existing endovascular modalities, but almost all RCTs compare only two modalities. At present, the optimal endovascular treatment for IPOD remains to be determined (9). To achieve a simultaneous comparison of all existing endovascular modalities, we included eligible RCTs in this network meta-analysis (NWM) to compare primary patency, target lesion revascularization (TLR), major amputation, and all-cause mortality among the different modalities. Meanwhile, AD + DCB was included for the first time in a network meta-analysis of the IPOD.

## Materials and methods

Our study followed the Preferred Reporting Items for Systematic Reviews and Meta-Analyses (PRISMA) guidelines (10) and was registered with the PROSPERO International Prospective Register of Systematic Reviews (CRD42022331626).

### Data sources and searches

We systematically searched Pubmed, Embase, and Cochrane databases on April 10, 2022. The search syntax included the following keywords: “randomized,” “stent,” “angioplasty,” “balloon,” “atherectomy,” “infrapopliteal,” “below-the-knee,” “crural,” “tibial,” “peroneal,” “critical limb ischemia,” and “chronic limb threatening ischemia.” The full search strategy was written in the Supplementary material. We also performed a gray literature search and examined the reference lists of the included studies and relevant reviews to identify other valuable articles.

The following selection criteria were employed to perform the analysis according to Population-Intervention-Comparison-Outcome-Study design (PICOS) principles. Population (P): patients underwent critical limb ischemia or intermittent claudication caused by IPOD. Intervention (I) and comparison (C): different endovascular modalities. Outcome (O): at least one accurate outcome of primary patency (6 or 12 months), TLR (6 or 12 months), major amputation (12 months), and all-cause death (12 months) was reported. Study design (S): RCTs published in English. When multiple articles were published by the same RCT, only one article containing the required data was included. RCTs involving additional drug therapy were excluded. RCTs involving additional devices that could have influenced the outcome, such as cutting balloons or lasers, were also excluded for lack of detailed data.

### Data extraction and quality assessment

After preliminary screening according to the title and abstract in the retrieval results, the articles that meet the inclusion criteria were identified by full text. The Cochrane Collaboration tool was used to assess the risk of bias for included RCTs (11). In addition to the outcome measures of efficacy and safety, the main data extracted is as follows: author, year, country, study design, treatment arms, and patients’ age, sex, and Rutherford category. Data search, data extraction, and bias assessment were performed independently by two investigators. Discrepancies were resolved by consensus, and a third investigator was consulted if necessary.

### Outcome measures

The main evaluation indicators included primary patency (6 and 12 months), defined as restenosis <50% as assessed by angiography or peak systolic velocity ratio (PSVR) < 2.4 as assessed by duplex ultrasound; TLR (6 and 12 months), defined as reintervention of target vessels through surgical bypass or endovascular therapy; major amputation (12 months), defined as amputation above the ankle; and all-cause mortality (12 months).

### Data analysis

To compare multiple endovascular modalities, we performed a frequentist NWM that can simultaneous analysis of both direct comparisons of treatments within studies and indirect comparisons of different treatments based on the same comparator (12). A random-effects model was used to allow for common heterogeneity across studies. BA was considered as the reference treatment modality in the comparison of different treatments. Before the meta-analysis, similarity and transitivity were assessed by comparing the clinical and methodological characteristics of the included RCTs.

Network maps for each outcome were constructed to show direct comparisons between different treatments in the included RCTs. The size of the nodes in the map was proportional to the sample size and the width of the line was proportional to the number of relevant studies. Inconsistency and node-splitting models were used to evaluate the consistency. A significant inconsistency was indicated when the *p*-value for inconsistency factors or the comparison between direct and indirect effects in the node-splitting analysis was < 0.05. The loop-specific method was also used to detect the inconsistency if there was a closed loop in the network map.

Analysis using a random-effects model produced results of pairwise comparisons, which were displayed in the league table and presented as risk ratios (RRs) and 95% confidence intervals (CIs). RR > 1 or < 1 favored one of two compared treatments over the other, whereas statistical significance was only indicated by the exclusion of 1 from 95% CIs. We generated surface under the cumulative ranking curve (SUCRA) plots and calculated the SUCAR values to rank the treatments involved in each outcome measure. In the analysis of one outcome measure, a large SUCRA value was equivalent to a better rank for a treatment. A “comparison-adjusted” funnel plot was structured to assess the small-study effects and publication bias in each meta-analysis. Patients in studies involving stents tend to have significantly shorter baseline lesion lengths, which may lead to potential heterogeneity in NWM. On the other hand, stents are currently mostly used as a rescue measure, rather than as a primary choice for IPOD. We therefore performed a subgroup analysis of the studies without primary stenting following the same procedure described above. All of the analyses were performed using Stata statistical software, version 16.0.

## Results

### Study selection

**FIGURE 1:**
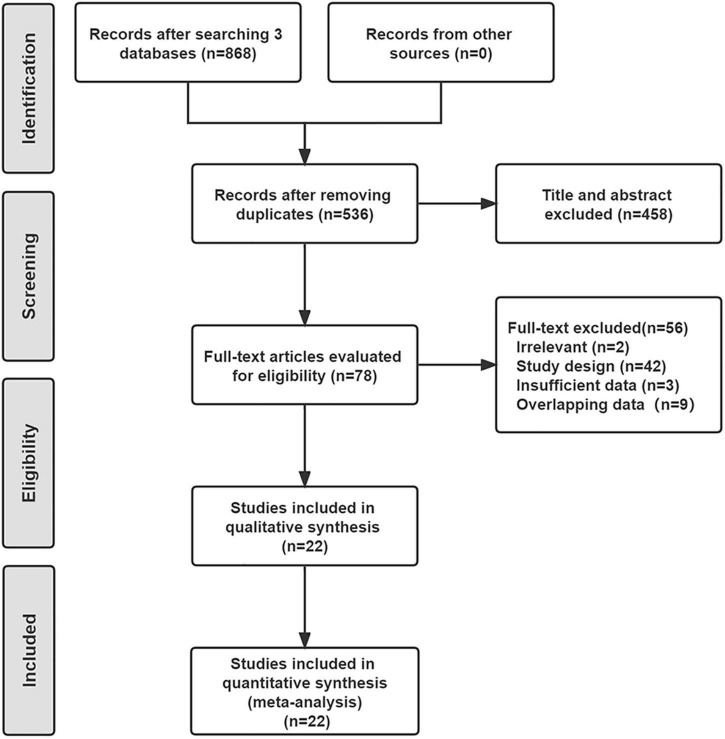
Flow chart of the eligible studies.

**FIGURE 2:**
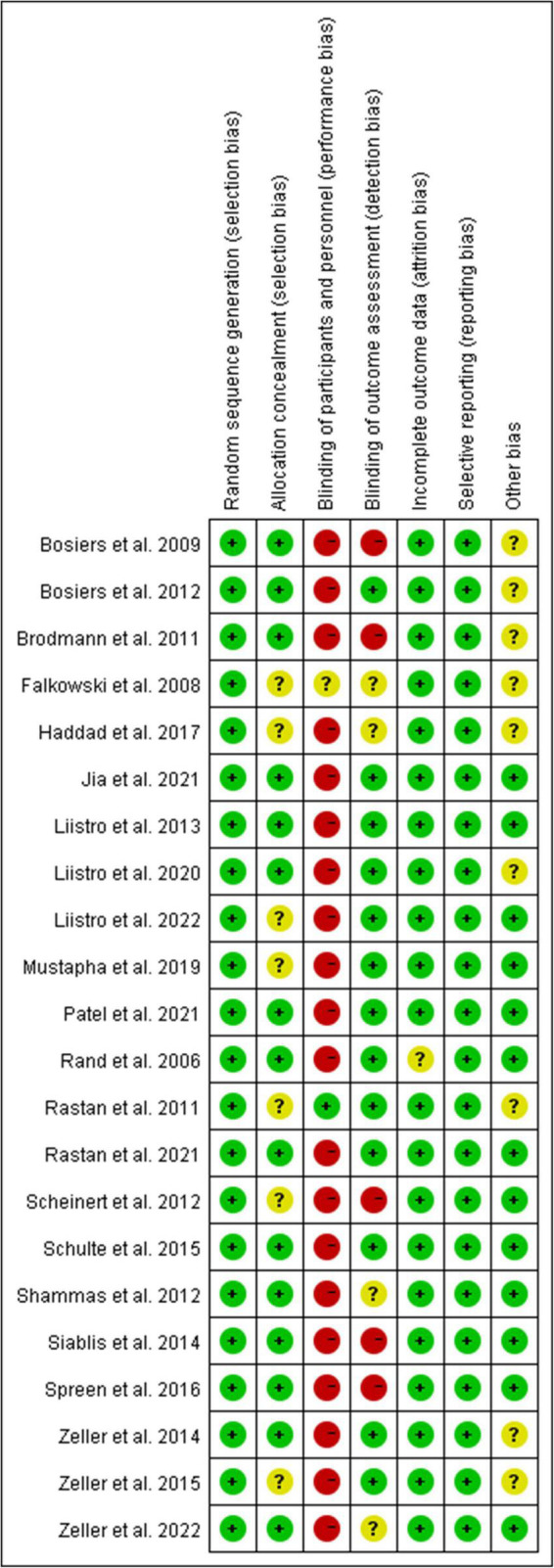
Analysis of the risk of bias according to the Cochrane Collaboration’s tool.

As shown in Figure 1, a total of 536 studies were found from three databases after removing duplications, and 22 double-arms RCTs (6–8, 13–31) that included 2,758 patients were eventually identified in this NWM. These studies compared nine endovascular modalities for the treatment of IPOD, including BA, AD + BA, DCB, AD + DCB, DES, balloon-expandable bare metal stent (BMS), self-expanding stent (SES), absorbable metal stents (AMS), and inorganics-coated stent (ICS). The characteristics and summary of eligible studies were detailed in Table 1, and the assessment of the risk of bias was shown in Figure 2. The high risk of bias was mainly concentrated in performance bias, which was not uncommon in the device trials in interventional cardiology. However, the included studies overall presented a low risk of bias.

**TABLE 1:** Characteristics of the eligible randomized controlled trials (RCT) included in the network meta-analysis.

RCT	Treatment arms	Enrolled patients	Age	Rutherford categories	Lesion length (mm)	Lesion stenosis (%)	Patency assessment	Follow-up* (year)
Zeller et al. (8)	AD + DCB vs DCB	32 vs 34	73.4 vs 76.5	3–5	101.3 vs 78.8	84.7 vs 81.8	DU	1
Liistro et al. (7)	DCB vs BA	23 vs 27	73.1 vs 69.6	4–5	215.4 vs 218.2	97.6 vs 96.3	DSA	0.75
Patel et al. (6)	DCB vs BA	70 vs 68	61 vs 64	4–6	90.3 vs 81.8	77.2 vs 78.8	DSA	1
Rastan et al. (14)	AD + DCB vs DCB	40 vs 40	71.5 vs 72.7	3–5	191.6 vs 160.8	86.8 vs 88.1	DU, DSA	1
Jia et al. (15)	DCB vs BA	61 vs 59	70.7 vs 70.8	3–6	170 vs 179.9	95 vs 97	DSA	1
Liistro et al. (7)	DCB vs BA	52 vs 53	75.4 vs 74.8	4–6	168 vs 187	91.5 vs 90.9	DSA	1
Mustapha et al. (16)	DCB vs BA	287 vs 155	72.9 vs 72.9	3–5	111.8 vs 94.7	86.7 vs 84.8	DU, DSA	0.5
Haddad et al. (17)	DCB vs BA	48 vs 45	52–74 vs 53–77	≥4	NM	NM	DU, DSA	1
Spreen et al. (18)	DES vs BA	73 vs 64	74.2 vs 72.9	4–6	21.1 vs 23.1	83.2 vs 83.1	CTA	10
Schulte et al. (20)	SES vs BA	45 vs 47	72.5 vs 73.3	3–5	34.1 vs 39.5	77.5 vs 75.1	DU, DSA	1
Zeller et al. (19)	DCB vs BA	36 vs 36	72.9 vs 69.6	2–5	113.1 vs 115	72.5 vs 72.1	DSA	1
Siablis et al. (22)	DES vs DCB	25 vs 25	75.3 vs 67.6	3–6	127 vs 148	86.8 vs 85.3	DSA	0.5
Zeller et al. (21)	DCB vs BA	239 vs 119	73.3 vs 71.7	3–6	101.5 vs 128.6	83.9 vs 86.6	DSA	5
Liistro et al. (23)	DCB vs BA	65 vs 67	74 vs 75	4–6	129 vs 131	97.2 vs 97.1	DU, DSA	5
Bosiers et al. (26)	BMS vs DES	66 vs 74	76 vs 75	4–5	18.9 vs 15.9	NM	DU, DSA	1
Scheinert et al. (25)	DES vs BA	99 vs 101	72.4 vs 74.3	3–5	26.9 vs 26.8	68.8 vs 74	DU, DSA	1
Shammas et al. (24)	AD + BA vs BA	25 vs 25	70.7 vs 71.8	4–6	NM	NM	NM	1
Brodmann et al. (28)	ICS vs BA	21 vs 33	68.9 vs 74.9	4–6	27.9 vs 78.5	NM	DU, DSA, MRA	1
Rastan et al. (27)	DES vs BMS	82 vs 79	73.4 vs 72.3	2–5	30 vs 31	88 vs 87	DU, DSA	3
Bosiers et al. (30)	BA vs AMS	57 vs 60	73.1 vs 74.7	4–5	NM	NM	DU, DSA	0.5
Falkowski et al. (29)	DES vs BMS	25 vs 25	68.3 vs 70.5	3–5	17.4 vs 18.2	NM	DSA	0.5
Rand et al. (33)	BA vs ICS	27 vs 24	72	Fontaine III-IV	24	NM	CTA, DSA	0.5

RCT, randomized controlled trial; BA, balloon angioplasty; DCB, drug-coated balloon; AD, atherectomy device; DES, drug-eluting stent; BMS, balloon-expandable bare metal stent; SES, self-expanding stent; AMS, absorbable metal stents; ICS, inorganics-coated stent; NM, not mentioned; DU, duplex ultrasound; DSA, digital substraction angiography; CTA, computed tomographic angiography; MRA, magnetic angiography. *This follow-up time is for the included trial, not for the included article.

### Network meta-analysis

#### Primary patency

**FIGURE 3:**
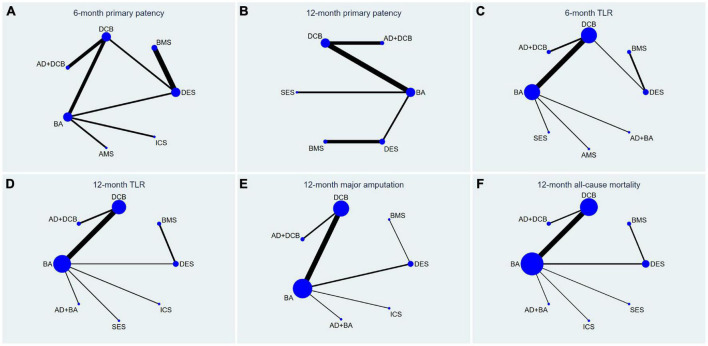
Network plots for all outcome measures. The size of the node was proportional to the sample size and the width of the line was proportional to the number of related studies. TLR, target lesion revascularization; BA, balloon angioplasty; DCB, drug-coated balloon; AD, atherectomy device; DES, drug-eluting stent; BMS, balloon-expandable bare metal stent; SES, self-expanding stent; AMS, absorbable metal stents; ICS, inorganics-coated stent. The first row is **(A–C)**, and the second row is **(D–F)**.

**FIGURE 4:**
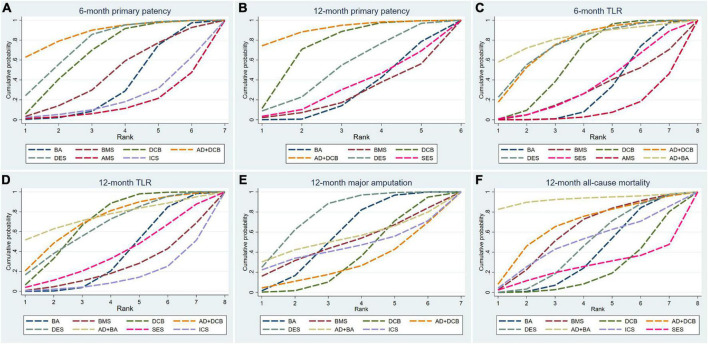
Surface under the cumulative ranking curve (SUCRA) plots for all outcome measures. TLR, target lesion revascularization; BA, balloon angioplasty; DCB, drug-coated balloon; AD, atherectomy device; DES, drug-eluting stent; BMS, balloon-expandable bare metal stent; SES, self-expanding stent; AMS, absorbable metal stents; ICS, inorganics-coated stent. The first row is **(A–C)**, and the second row is **(D–F)**.

The results of 6-month primary patency (restenosis <50%) rates were reported in eleven studies (6–8, 14, 18, 22, 26, 27, 29–31) involving seven endovascular modalities (Figure 3A). No significant inconsistency was found by node-splitting analysis (all *p*-value > 0.326). The loop-specific analysis (BA-DCB-DES) also indicated no significant inconsistency (inconsistency factor (IF) = 1.68; 95% CI 0.00, 4.24; *p* = 0.00). As shown in Table 2, the analysis did not produce significant results for the 6-month primary patency in any of the comparisons. The SUCRA plot is shown in Figure 4A and the values are listed as follows: AD + DCB (SUCRA = 87.5), DES (SUCRA = 76.6), DCB (SUCRA = 67.6), BMS (SUCRA = 46.2), BA (SUCRA = 35.4), ICS (SUCRA = 21.7), AMS (SUCRA = 15.1; Table 3). The comparison-adjusted funnel plot for 6-month primary patency is presented in Supplementary Figure 1A. We found significant visual asymmetry, indicating the possibility of publication bias or a small-study effect.

**TABLE 2:** Comparison results of network meta-analysis on 6-month (left) and 12-month (right) primary patency.

AD + DCB	0.45 (0.11, 1.83)	0.68 (0.30, 1.50)	0.31 (0.06, 1.47)	**0.34 (0.12, 0.93)**	–	–	0.34 (0.08, 1.37)
1.40 (0.37, 5.29)	DES	1.50 (0.47, 4.79)	0.68 (0.34, 1.36)	0.75 (0.28, 1.99)	–	–	0.75 (0.19, 2.97)
1.57 (0.63, 3.90)	1.12 (0.43, 2.95)	DCB	0.45 (0.12, 1.75)	**0.50 (0.27, 0.93)**	–	–	0.50 (0.16, 1.58)
2.21 (0.50, 9.90)	1.58 (0.79, 3.15)	1.41 (0.43, 4.63)	BMS	1.10 (0.33, 3.64)	–	–	1.10 (0.24, 5.14)
2.78 (0.83, 9.30)	1.98 (0.77, 5.11)	1.77 (0.80, 3.92)	1.25 (0.39, 4.04)	BA	–	–	1.00 (0.38, 2.64)
4.16 (0.74, 23.23)	2.96 (0.63, 13.96)	2.65 (0.61, 11.40)	1.88 (0.35, 10.21)	1.50 (0.44, 5.10)	ICS	–	–
5.06 (0.90, 28.53)	3.61 (0.76, 17.15)	3.22 (0.74, 14.02)	2.28 (0.42, 12.53)	1.82 (0.53, 6.28)	1.22 (0.21, 6.94)	AMS	–
–	–	–	–	–	–	–	SES

BA, balloon angioplasty; DCB, drug-coated balloon; AD, atherectomy device; DES, drug-eluting stent; BMS, balloon-expandable bare metal stent; SES, self-expanding stent; AMS, absorbable metal stents; ICS, inorganics-coated stent. Bold values represent statistically significant values and gray grids represent treatment modalities.

**TABLE 3:** Surface under the cumulative ranking curve values of endovascular modalities for all terms of analysis.

6-m primary patency		12-m primary patency		6-m TLR		12-m TLR		12-m major amputation		12-m all-cause mortality	
AD + DCB	87.5	AD + DCB	91	AD + BA	83.1	AD + BA	75.8	DES	78.6	AD + BA	92.5
DES	76.6	DCB	73.6	AD + DCB	75.4	AD + DCB	71.7	BA	57.8	AD + DCB	66
DCB	67.6	DES	52.1	DES	74.7	DCB	70.6	AD + BA	54.3	BMS	60.2
BMS	46.2	SES	31.9	DCB	60.3	DES	66.1	BMS	49.5	ICS	49.9
BA	35.4	BA	27.1	SES	35.5	SES	38.9	ICS	45.2	DES	46.4
ICS	21.7	BMS	24.2	BA	30.8	BA	37	DCB	35.7	BA	38.8
AMS	15.1			BMS	29.4	BMS	25.2	AD + DCB	28.8	SES	23.9
				AMS	10.8	ICS	14.7			DCB	22.3

RCT, randomized controlled trial; BA, balloon angioplasty; DCB, drug-coated balloon; AD, atherectomy device; DES, drug-eluting stent; BMS, balloon-expandable bare metal stent; SES, self-expanding stent; AMS, absorbable metal stents; ICS, inorganics-coated stent; NM, not mentioned.

The results of 12-month primary patency (restenosis <50%) rates were reported in nine studies (8, 14, 17, 20, 21, 23, 25–27) involving six endovascular modalities (Figure 3B). No significant inconsistency was found by node-splitting analysis (all *p*-value > 0.995). The loop-specific analysis found no closed loops. BA had a lower 12-month primary patency rate than DCB (RR 0.50, CI 0.27, 0.93) and AD + DCB (RR 0.34, CI 0.12, 0.93). No significant results were identified in the other comparisons (Table 2). The SUCRA plot is shown in Figure 4B and the values are listed as follows: AD + DCB (SUCRA = 91), DCB (SUCRA = 73.6), DES (SUCRA = 52.1), SES (SUCRA = 31.9), BA (SUCRA = 27.1), BMS (SUCRA = 24.2; Table 3). The comparison-adjusted funnel plot for 12-month primary patency is shown in Supplementary Figure 1B, with no significant visual asymmetry observed, indicating no evidence of the publication bias and small-study effect.

#### Target lesion revascularization

The results of 6-month TLR rates were reported in fourteen RCTs (6–8, 13–16, 19, 20, 22, 24, 26, 29, 30) involving eight endovascular modalities (Figure 3C). No significant inconsistency was found by node-splitting analysis (all *p*-value > 0.999), and no closed loop needed loop-specific analysis. AD + DCB significantly decreased 6-month TLR compared with AMS (RR 0.15, CI 0.03, 0.90), and DES significantly decreased it compared with BMS (RR 0.25, CI 0.09, 0.71). DCB had a lower 6-month TLR rate than AMS (RR 0.26, CI 0.08, 0.86) and BA (RR 0.51, CI 0.30, 0.89). No significant results were identified in the other comparisons (Table 4). The SUCRA plot for 6-month TLR is shown in Figure 4C. AD + BA had the highest SUCRA values (SUCRA = 83.1), followed by AD + DCB (SUCRA = 75.4), DES (SUCRA = 74.7), DCB (SUCRA = 60.3), SES (SUCRA = 35.5), BA (SUCRA = 30.8), BMS (SUCRA = 29.4), AMS (SUCRA = 10.8; Table 3). No significant visual asymmetry was observed in the comparison-adjusted funnel plot for 6-month TLR (Supplementary Figure 1C).

**TABLE 4:** Comparison results of network meta-analysis on 6-month (left) and 12-month (right) target lesion revascularization.

AD + BA	1.53 (0.11, 20.42)	1.82 (0.13, 26.19)	1.70 (0.15, 18.84)	3.11 (0.22, 44.34)	3.00 (0.29, 31.49)	4.45 (0.26, 74.69)	–	6.06 (0.42, 87.39)
0.45 (0.02, 12.78)	AD + DCB	1.19 (0.23, 6.26)	1.11 (0.42, 2.92)	2.03 (0.39, 10.55)	1.96 (0.66, 5.80)	2.90 (0.43, 19.42)	–	3.96 (0.75, 20.92)
0.45 (0.01, 16.62)	1.01 (0.10, 10.27)	DES	0.93 (0.24, 3.61)	1.71 (0.29, 9.97)	1.65 (0.47, 5.79)	2.44 (0.97, 6.15)	–	3.33 (0.56, 19.75)
0.25 (0.01, 5.48)	0.57 (0.15, 2.18)	0.56 (0.08, 3.75)	DCB	1.82 (0.48, 6.95)	**1.76 (1.07, 2.90)**	2.61 (0.51, 13.42)	–	3.56 (0.92, 13.83)
0.14 (0.01, 3.56)	0.31 (0.05, 2.03)	0.30 (0.03, 3.08)	0.54 (0.14, 2.05)	SES	0.97 (0.28, 3.34)	1.43 (0.20, 10.50)	–	1.95 (0.33, 11.45)
0.13 (0.01, 2.68)	0.29 (0.07, 1.24)	0.29 (0.04, 2.08)	**0.51 (0.30, 0.89)**	0.95 (0.28, 3.23)	BA	1.48 (0.31, 7.05)	–	2.02 (0.57, 7.14)
0.11 (0.00, 4.87)	0.26 (0.02, 3.24)	**0.25 (0.09, 0.71)**	0.45 (0.05, 3.88)	0.84 (0.07, 10.54)	0.88 (0.09, 8.09)	BMS	–	1.36 (0.18, 10.13)
0.07 (0.00, 1.64)	**0.15 (0.03, 0.90)**	0.15 (0.02, 1.39)	**0.26 (0.08, 0.86)**	0.49 (0.10, 2.44)	0.51 (0.18, 1.46)	0.59 (0.05, 6.86)	AMS	–
–	–	–	–	–	–	–	–	ICS

BA, balloon angioplasty; DCB, drug-coated balloon; AD, atherectomy device; DES, drug-eluting stent; BMS, balloon-expandable bare metal stent; SES, self-expanding stent; AMS, absorbable metal stents; ICS, inorganics-coated stent. Bold values represent statistically significant values and gray grids represent treatment modalities.

The results of 12-month TLR rates were also reported in fourteen RCTs (6–8, 14, 15, 17, 19–21, 24–28) involving eight endovascular modalities (Figure 3D). No significant inconsistency was found by node-splitting analysis (all *p*-value > 0.999), and the loop-specific analysis found no loops. We found BA had a higher 12-month TLR rate than DCB (RR 1.76, CI 1.07, 2.90), while no significant differences were identified among the other comparisons (Table 4). The SUCRA plot for 12-month TLR is shown in Figure 4D. AD + BA still had the highest SUCRA values (SUCRA = 75.8), followed by AD + DCB (SUCRA = 71.7), DCB (SUCRA = 70.6), DES (SUCRA = 66.1), SES (SUCRA = 38.9), BA (SUCRA = 37), BMS (SUCRA = 25.2), ICS (SUCRA = 14.7; Table 3). The comparison-adjusted funnel plot for 12-month TLR was visually symmetrical, suggesting no evidence of the publication bias and small-study effect (Supplementary Figure 1D).

#### Major amputation

Fourteen studies (6–8, 14, 15, 17–19, 21, 23–25, 27, 28) reported the results of seven endovascular modalities for 12-month major amputations (Figure 3E). The node-splitting analysis did not yield significant results (all *p*-value > 0.999), and the loop-specific analysis found no closed loop. We did not find any endovascular modality that had a significantly lower rate of 12-month major amputation compared to the others (Table 5). The SUCRA plot for 12-month major amputation is presented in Figure 4E, and the SUCRA values are as follows: DES (SUCRA = 78.6), BA (SUCRA = 57.8), AD + BA (SUCRA = 54.3), BMS (SUCRA = 49.5), ICS (SUCRA = 45.2), DCB (SUCRA = 35.7) and AD + DCB (SUCRA = 28.8; Table 3). The comparison-adjusted funnel plot for 12-month major amputation is shown in Supplementary Figure 1E, with a visual symmetry observed, indicating no evidence of publication bias and small-study effect.

**TABLE 5:** Comparison results of network meta-analysis on 12-month major amputation (left) and 12-month all-cause mortality (right).

DES	1.11 (0.69, 1.78)	0.11 (0.01, 1.94)	0.84 (0.50, 1.41)	0.92 (0.24, 3.55)	1.33 (0.72, 2.43)	0.68 (0.20, 2.26)	1.74 (0.29, 10.57)
0.63 (0.31, 1.30)	BA	0.10 (0.01, 1.68)	0.75 (0.37, 1.53)	0.82 (0.23, 2.94)	1.19 (0.82, 1.74)	0.61 (0.20, 1.85)	1.57 (0.27, 8.94)
0.63 (0.01, 31.88)	1.00 (0.02, 47.18)	AD + BA	7.34 (0.41, 131.49)	8.04 (0.37, 173.65)	11.65 (0.69, 195.98)	5.94 (0.29, 120.53)	15.27 (0.57, 412.26)
0.51 (0.05, 5.46)	0.80 (0.07, 9.63)	0.80 (0.01, 78.80)	BMS	1.10 (0.26, 4.68)	1.59 (0.71,3.53)	0.81 (0.22, 3.02)	2.08 (0.32, 13.62)
0.41 (0.01, 21.22)	0.65 (0.01, 31.43)	0.65 (0.00, 153.80)	0.80 (0.01, 80.73)	ICS	1.45 (0.39, 5.45)	0.74 (0.14, 3.99)	1.90 (0.22, 16.39)
0.42 (0.17, 1.03)	0.67 (0.39, 1.13)	0.67 (0.01, 32.64)	0.83 (0.07, 10.50)	1.03 (0.02, 51.91)	DCB	0.51 (0.18, 1.45)	1.31 (0.22, 7.79)
0.26 (0.03, 2.47)	0.41 (0.05, 3.48)	0.41 (0.01, 33.68)	0.51 (0.02, 13.50)	0.64 (0.01, 53.38)	0.62 (0.08, 4.88)	AD + DCB	2.57 (0.33, 20.30)
-	-	-	-	-	-	-	SES

BA, balloon angioplasty; DCB, drug-coated balloon; AD, atherectomy device; DES, drug-eluting stent; BMS, balloon-expandable bare metal stent; SES, self-expanding stent; AMS, absorbable metal stents; ICS, inorganics-coated stent. Gray grids represent treatment modalities.

#### All-cause mortality

Seventeen studies (6–8, 13–15, 17–21, 23–28) reported the results of eight endovascular modalities for 12-month all-cause mortality (Figure 3F). The node-splitting analysis did not yield significant results (all *p*-value > 0.999), and the loop-specific analysis found no closed loop. Analysis showed no significant difference in 12-month all-cause mortality among these eight endovascular modalities (Table 5). The SUCRA plot for 12-month major amputation is presented in Figure 4F, and the SUCRA values are as follows: AD + BA (SUCRA = 92.5), AD + DCB (SUCRA = 66), BMS (SUCRA = 60.2), ICS (SUCRA = 49.9), DES (SUCRA = 46.4), BA (SUCRA = 38.8), SES (SUCRA = 23.9) and DCB (SUCRA = 22.3; Table 3). The comparison-adjusted funnel plot for 12-month all-cause mortality was visually symmetrical, suggesting no evidence of publication bias and small-study effect (Supplementary Figure 1F).

#### Subgroup analysis

A total of 12 studies were included in the subgroup analyses (6–8, 13–17, 19, 21, 23, 24). Subgroup analyses of primary patency included three endovascular modalities (BA, DCB, AD + DCB), and subgroup analyses of other outcome measures included four modalities (BA, AD + BA, DCB, AD + DCB). The results of pairwise comparisons changed slightly. The reduced 12-month patency rate of BA compared with AD + DCB (RR 0.33, CI 0.10, 1.07) and DCB (RR 0.49, CI 0.24, 1.01) approached but did not reach a statistical difference, which may be related to the sample size not being sufficiently large in the subgroup analysis (Supplementary Figure 2). While the 6 and 12-month TLR rates of DCB were still significantly lower than those of BA. The SUCRA ranking of these treatment modalities involved in the subgroup analyses did not change for any of the outcome measures (Supplementary Figure 3).

## Discussion

Individual clinical and anatomical factors determine whether IPOD patients receive endovascular intervention or bypass surgery. The Global Vascular Guideline recommends endovascular intervention be preferred for high-risk patients because of the reduced complication rate (31). A recent IPOD study reported no difference in amputation-free survival and overall survival with endovascular treatment compared with bypass surgery, but significant reductions in major amputation and death (32). To compare the advantages of different endovascular modalities in IPOD, we performed an updated NWM for all eligible RCTs. Although some RCTs involving SES (20), ICS (28, 33), and AMS (30) were only conducted many years ago, meaning that these modalities do not show significant advantages to become mainstream treatments, their inclusion of them could produce a more complete network of endovascular modalities for treating IPOD.

None of the modalities showed significantly different primary patency rates at 6 months, but both AD + DCB and DCB had significantly higher primary patency rates than BA at 12 months. These two positive outcomes were weakened by subgroup analyses, most likely because the sample size was reduced. In addition, AD + DCB had the highest SUCRA values at these two time points, meaning that AD + DCB was the most effective treatment in terms of primary patency, followed by DCB and DES. Since the only RCT including the AD + BA arm did not provide the data of primary patency, AD + BA could not participate in the comparison of primary patency. AMS significantly increased 6-month TLR compared with AD + DCB and DCB, and BMS significantly increased compared with DES. DCB’s 6 and 12-month TLR rates were significantly lower than BA’s. In terms of TLR, AD + BA was considered the most effective treatment at the 6 and 12-month follow-up, followed by AD + DCB, DCB, and DES, with consistent order in primary patency. Previous network meta-analysis found that DCB was superior to AD + BA in terms of 12-month TLR (34), and the reason for this inconsistency may be that we included newer RCTs that included the DCB arm (6–8, 13–16, 22). Similar to the results reported by Zhou et al., we also found that DCB was better than DES in terms of primary patency and TLR at 12 months (34), but we also found that the result was reversed at 6 months, suggesting that the advantages of DCB in IPOD may be shown in the long-term. There were no significant differences between the different modalities of 12-month major amputation and 12-month all-cause mortality. However, DES was the safest for major amputation, which was the same as the previous study (34), and AD + BA was the safest for all-cause death according to the SUCRA values. It is worth noting that AD + DCB and DCB had the lowest SUCRA values for 12-month major amputations, suggesting that they had the worst safety in this term. ICS, AMS, and SES did not show a significant advantage in the analysis of these outcome measures, nor did they achieve higher SUCRA values, which is consistent with their lack of widespread clinical application in IPOD. Overall, except for 12-month major amputations, AD combined with BA or DCB always showed higher SUCRA values than a single modality, suggesting that combination therapy may be a better treatment strategy. Moreover, AD + BA invariably ranks higher than AD + DCB in comparisons including these two combinations.

To inhibit the intimal hyperplasia response to the barotrauma during endovascular revascularization, antiproliferative drugs were used to upgrade balloons or stents (2). In our NWM, except for one included DES study that used paclitaxel-eluting stents, five other DES studies used sirolimus (or its analogue) eluting stents, while all DCB studies used paclitaxel-coated balloons. Paclitaxel-coated balloons did improve primary patency and TLR, but their outcomes in terms of amputation were unsatisfactory. Katsanos et al. found that paclitaxel-coated balloons significantly worsened amputation-free survival in IPOD and significantly increased the risk of major amputation in peripheral artery disease compared with BA (35, 36), which supported our finding that AD + DCB and DCB had the lowest SUCRA values for 12-month major amputation. A significant dose-dependent relationship was detected between the dose of perioperative paclitaxel exposure and the risk of major amputation, implying that high-dose paclitaxel devices increased the risk of major amputation more significantly. This phenomenon is most likely due to distal embolization and prolonged tissue residence of large amounts of escaped cytotoxic solid paclitaxel (36, 37). In terms of DES, a meta-analysis of DES in the treatment of IPOD found that the primary patency of sirolimus and its analogs eluting stents was significantly higher than that of paclitaxel-eluting stents, and a similar trend was also observed for the outcome of TLR (38). DES shows potential benefit in short infrapopliteal lesions, but the lesions leading to lower limb ischemia are often extensive. The Global Vascular Guideline suggested that DES is more commonly used as endovascular “bailout” in short and proximal infrapopliteal lesions.

During endovascular treatment, atherectomy is a common method for vessel preparation, which reduces the risk of dissection and rupture, maximizes luminal gain, and facilitates the outcomes of endovascular interventions (2). Currently, there are four main types of ADs available, including: orbital, rotational, directional, and laser, but only three RCTs involving orbital atherectomy (OA) or directional atherectomy (DA) for IPOD have been published. RCTs of Zeller et al. and Rastan et al. reported that the efficacy and safety of adjunctive ADs (OA and DA) were similar rather than significantly better than that of DCB alone, and inadequate sample size may be an important reason for limiting the significant advantage of ADs (8, 14). However, Zeller et al. also found that the 6 and 12-month primary patency of OA + DCB were numerically higher (*p*-value = 0.065, 0.076), which showed a trend of statistical difference. Shammas et al. indicated that the OA + BA arm had significantly lower 12-month all-cause mortality than the BA arm in the treatment of IPOD (24). Of course, several non-randomized studies have also demonstrated the utility of ADs for IPOD patients, including rotational and laser atherectomy (39–42). On the other hand, there is a view that vessel preparation with ADs achieves high technical success of endovascular treatment by increasing the risk of perioperative distal embolization (43). This seems to match precisely with our results that AD + DCB had a lower rank than DCB for 12-month major amputations and was the lowest rank. A retrospective study found that AD with angioplasty was associated with higher reintervention and local complications compared with angioplasty alone (44). Although the use of ADs on IPOD remains controversial due to the lack of updated RCTs to reveal more positive comparative results, our NWM supported adjunctive ADs had the best rank in terms of primary patency, TLR, and all-cause mortality, particularly AD + BA, which was also the third most effective for 12-month major amputation.

Our NWM has some limitations. First, only RCTs that met the criteria were included in this NWM, so the number of included studies for some endovascular modalities may be low, or even only one, which could weaken the results. Second, not all studies included some patients with intermittent claudication, and whether this would cause significant heterogeneity needs further investigation. Third, both OA + DCB and DA + DCB had only one eligible RCT and the sample size was not very large, so they were combined into AD + DCB for analysis rather than independently participating in the NWM. Since OA and DA achieve similar work (2), we consider this combination is acceptable. Currently, data on the treatment of IPOD whit ADs is still limited and more high-quality RCTs are urgently needed.

In conclusion, this NWM found that ADs showed noteworthy advantages in multiple terms for IPOD except for 12-month major amputation rate. AD + BA may be a better treatment option for IPOD than AD + DCB. AD + DCB appears to be the most effective in terms of 6 and 12-month primary patency, while AD + BA was the most effective in terms of 6 and 12-month TLR and was the safest in terms of 12-month all-cause mortality. The 12-month major amputation rates of DES may be superior to those of other modalities, while DCB and AD + DCB seem to be less safe. The efficacy and safety of ADs deserves further investigation.

## Data availability statement

The original contributions presented in this study are included in the article/Supplementary material, further inquiries can be directed to the corresponding authors.

## Author contributions

JG and YN contributed to the study design and interpretation of the results. JG, ZS, SW, and FZ collected the data. JG, HW, and YL analyzed the data and prepared the figures. JG prepared the manuscript. YN, LG, and YG revised the manuscript. All authors contributed to the article and approved the submitted version.
