# Supplementary material for: The efficacy and safety of different endovascular modalities for infrapopliteal arteries lesions: A network meta-analysis of randomized controlled trials
Source: Front Cardiovasc Med. 2022 Nov 9;9:993290. doi: 10.3389/fcvm.2022.993290 (PMC9682151; doi:10.3389/fcvm.2022.993290)
Supplement: Supplementary file 1 [file Data_Sheet_1.docx]

Supplementary Material

## Search strategy:

Pubmed: ("randomized"[Title/Abstract] OR "randomised"[Title/Abstract]) AND ("stent*"[Title/Abstract] OR "angioplasty"[Title/Abstract] OR "balloon*"[Title/Abstract] OR "atherectomy"[Title/Abstract]) AND ("infrapopliteal"[Title/Abstract] OR "below the knee"[Title/Abstract] OR "crural"[Title/Abstract] OR "tibial"[Title/Abstract] OR " peroneal "[Title/Abstract] OR "critical limb ischaemia"[Title/Abstract] OR "chronic limb threatening ischemia" [Title/Abstract])

Embase：(randomized:ab,ti OR randomised:ab,ti) AND (stent*:ab,ti OR angioplasty:ab,ti OR balloon*:ab,ti OR atherectomy:ab,ti) AND (infrapopliteal:ab,ti OR 'below the knee':ab,ti OR crural:ab,ti OR tibial:ab,ti OR ' Peroneal':ab,ti OR 'critical limb ischaemia':ab,ti OR 'chronic limb threatening ischemia':ab,ti)

Cochrane: ((randomized):ti,ab,kw OR (randomised):ti,ab,kw) AND ((stent*):ti,ab,kw OR (angioplasty):ti,ab,kw OR (balloon*):ti,ab,kw OR (atherectomy):ti,ab,kw) AND ((infrapopliteal):ti,ab,kw OR (below the knee):ti,ab,kw OR (crural):ti,ab,kw OR (tibial):ti,ab,kw OR (peroneal):ti,ab,kw OR (critical limb ischaemia):ti,ab,kw OR (chronic limb threatening ischemia):ti,ab,kw)

## Supplementary Figures


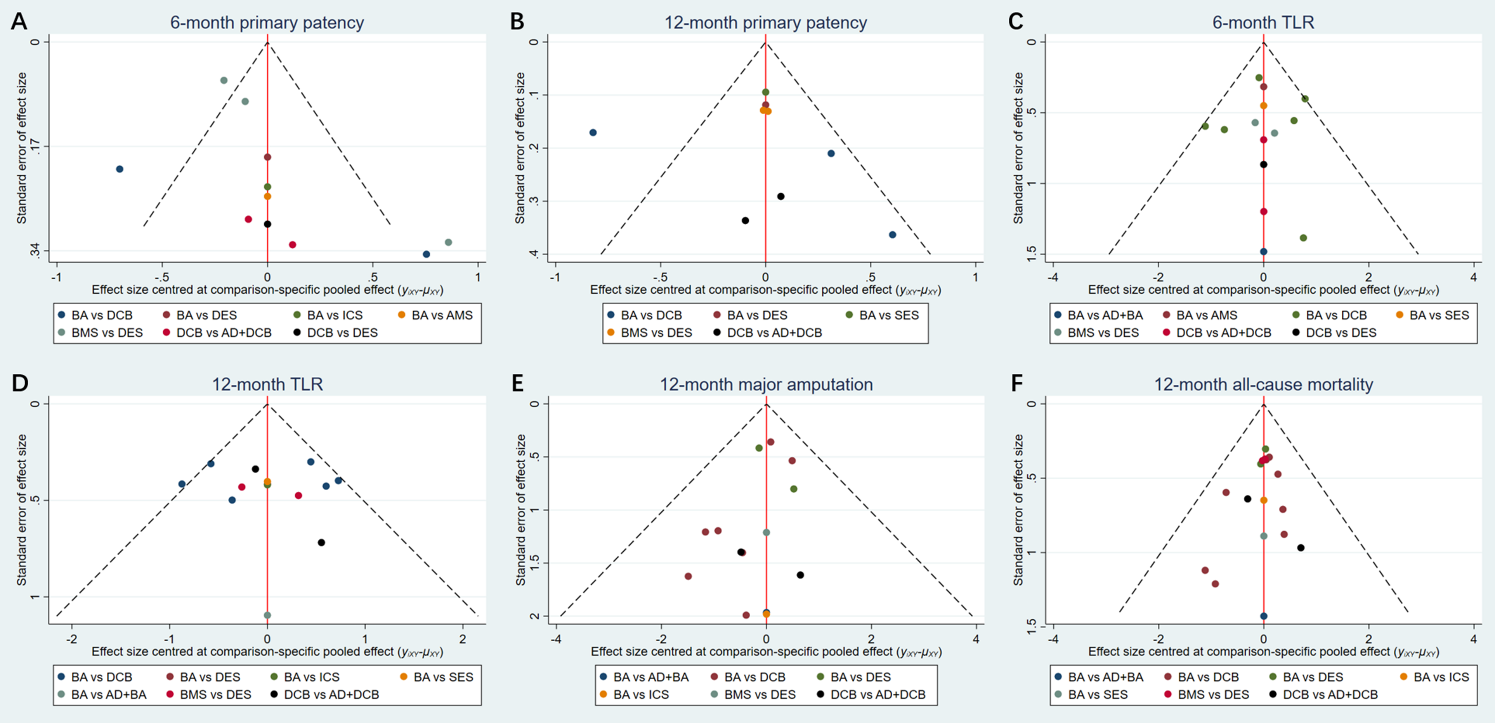


**Supplementary Figure 1.** Comparison-adjusted funnel plot for all outcome measures. BA: balloon angioplasty; DCB: drug-coated balloon; AD: atherectomy device; DES: drug-eluting stent; BMS: balloon-expandable bare metal stent; SES: self-expanding stent; AMS: absorbable metal stents; ICS: inorganics-coated stent.


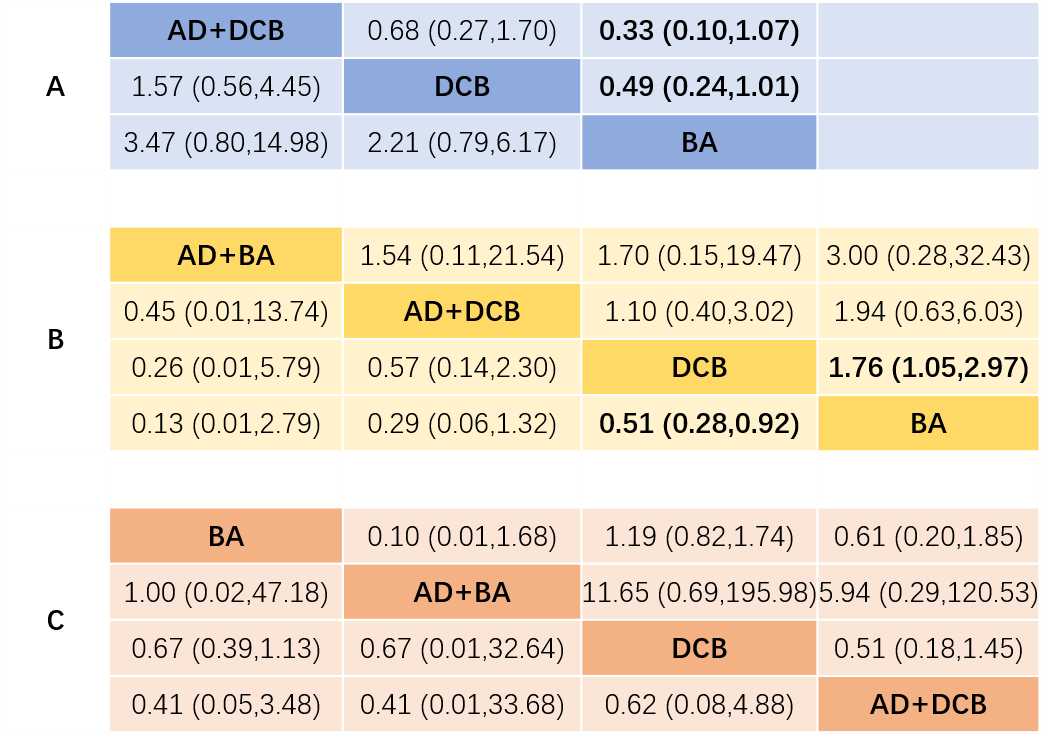


**Supplementary Figure 2.** Comparison results of subgroup analysis. A, 6-month primary patency (left) and 12-month primary patency (right). B, 6-month target lesion revascularization (left) and 12-month target lesion revascularization (right); C, 12-month major amputation (left) and 12-month all-cause mortality. BA: balloon angioplasty; DCB: drug-coated balloon; AD: atherectomy device.


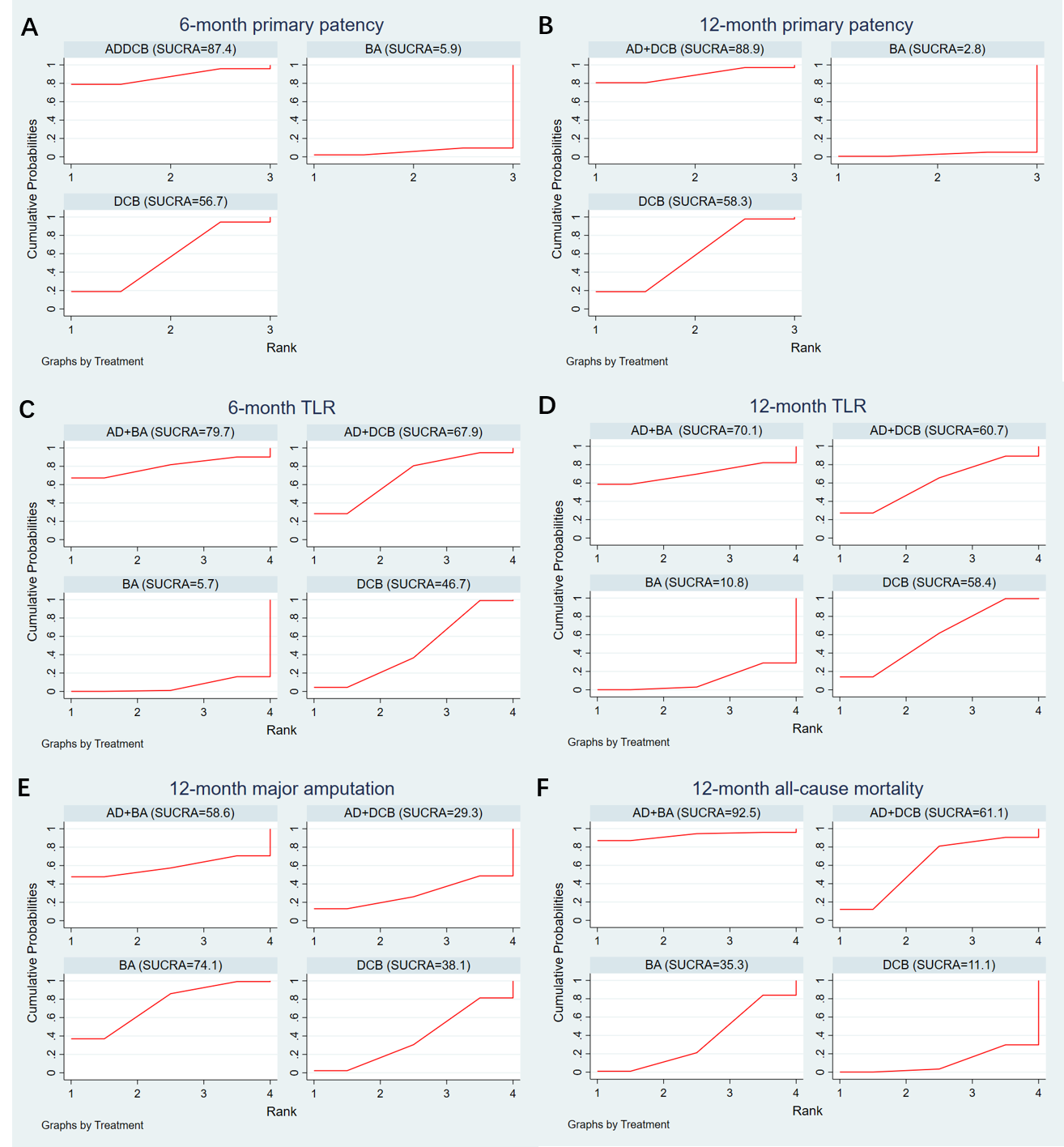


**Supplementary Figure 3.** Surface under the cumulative ranking curve (SUCRA) plots for subgroup analysis. TLR: target lesion revascularization; BA: balloon angioplasty; DCB: drug-coated balloon; AD: atherectomy device.
